# Copeptin and the risk of incident stroke, CHD and cardiovascular mortality in older men with and without diabetes: The British Regional Heart Study

**DOI:** 10.1007/s00125-016-4011-7

**Published:** 2016-06-16

**Authors:** S. Goya Wannamethee, Paul Welsh, Lucy Lennon, Olia Papacosta, Peter H. Whincup, Naveed Sattar

**Affiliations:** 1 Department of Primary Care and Population Health, UCL Medical School, Royal Free Campus, Rowland Hill St, London, NW3 2PF UK; 2 Institute of Cardiovascular and Medical Sciences, BHF Glasgow Cardiovascular Research Centre, University of Glasgow, Glasgow, UK; 3 Population Health Research Institute, St George’s, University of London, London, UK

**Keywords:** Cardiovascular mortality, Copeptin, Coronary heart disease, Diabetes, Epidemiology, Stroke

## Abstract

**Aims/hypothesis:**

This study aimed to examine the association between copeptin (a surrogate marker of arginine vasopressin) and incident stroke, CHD and cardiovascular mortality in older men with and without diabetes.

**Methods:**

We conducted a prospective study of 3536 men aged 60–79 years who were followed for an average of 13 years. During this period, there were 437 major CHD events (fatal and non-fatal myocardial infarction [MI]), 323 stroke events (fatal and non-fatal) and 497 cardiovascular disease (CVD) deaths. Prevalent diabetes was defined on the basis of a history of doctor-diagnosed diabetes or fasting blood glucose ≥7.0 mmol or HbA_1c_ ≥6.5% (48 mmol/mol) (*n* = 428).

**Results:**

No association was seen between copeptin and incident stroke or CVD mortality in men without diabetes after adjustment for conventional cardiovascular risk factors, renal dysfunction, and insulin and N-terminal pro B-type natriuretic peptide levels. In contrast, elevated copeptin levels were associated with an increased risk of stroke and CVD mortality in men with diabetes after these adjustments. Compared with those in the lowest tertile of copeptin, men in the top tertile had adjusted relative HRs for stroke and CVD death of 2.34 (95% CI 1.04, 5.27) and 2.21 (1.12, 4.36), respectively. The risk of stroke and CVD mortality remained increased after the exclusion of men with prevalent stroke or MI. Higher levels of copeptin were associated with increased risk of CHD in the diabetic and non-diabetic groups, but these associations were attenuated after exclusion of individuals with a previous stroke or MI.

**Conclusions/interpretation:**

Copeptin was independently associated with an increased risk of incident stroke and CVD mortality in men with diabetes, but not in men without diabetes. Targeting the arginine vasopressin system might have beneficial effects on CVD mortality and stroke risk in older men with diabetes.

## Introduction

Cardiovascular disease (CVD) is the leading cause of death in patients with type 2 diabetes mellitus [1, 2]. It is well established that type 2 diabetes is a major risk factor for CVD, including myocardial infarction (MI) and stroke [1–3]. Type 2 diabetes has been associated with a two- to sixfold increase in the risk of stroke compared with no diabetes [3]. The reduction of cardiovascular risk in individuals with type 2 diabetes is thus of paramount importance.

Arginine vasopressin (AVP), also known as antidiuretic hormone, is a nonapeptide that is produced in the hypothalamus. AVP is released from the neurohypophysis to promote renal water conservation, and plays important roles in the regulation of the cardiovascular system, water electrolyte balance and many functions of the central nervous system [4, 5]. Vasopressin receptors are widely distributed throughout the brain [6]. They are present in neurons, astrocytes and their perivascular processes, blood vessel endothelial and smooth muscle cells, and the choroid plexus. These locations suggest that vasopressin may participate in regulating vascular resistance in the cerebral circulation and water homeostasis in the brain [6]. Human and animal studies have indicated that the AVP system may play a role in glucose homeostasis, insulin resistance, and lipid and fat metabolism [7–9], and vasopressin has been reported to be elevated in patients with type 2 diabetes [10]. However, plasma AVP is difficult to measure because of its instability and short half-life [11]. Plasma carboxy-terminal pro-vasopressin (copeptin), the C-terminal part of the AVP precursor peptide, is stable in serum and plasma, making it a more convenient biomarker. Indeed, copeptin has been established as a reliable marker of the circulating AVP concentration in routine clinical practice [11].

Copeptin is known to be raised in individuals with type 2 diabetes and has been associated with the development of diabetes [12, 13]. Raised copeptin has also been associated with CVD mortality in patients with type 2 diabetes complicated by end-stage renal disease or acute MI, as well as in individuals with diabetes who are treated in primary care and those in the general population [14–18]. Stroke incidence rises steeply with age and the number of older people with type 2 diabetes is increasing. Attention has therefore turned to identifying new therapeutic approaches to preventing stroke in individuals with type 2 diabetes [19]. Copeptin has been shown to predict incident stroke in diabetic patients undergoing haemodialysis [15], but its ability to predict incident stroke events in individuals with and without diabetes among the older general population has not yet been assessed. To evaluate these issues, we examined the association between copeptin and incident stroke, as well as CHD events (fatal and non-fatal) and CVD mortality, in men with and without diabetes.

## Methods

### Participants

The British Regional Heart Study is a prospective study of CVD involving 7735 men aged 40–59 years drawn from general practice in each of 24 British towns, with participants recruited between 1978 and 1980 [20]. The population is socioeconomically representative of British men, but is predominantly comprised of white Europeans (>99%). In 1998–2000, all of the surviving men, then aged 60–79 years (mean age 68.7 years), were invited for a 20th year follow-up examination. The men completed a questionnaire, which included questions on their medical history and lifestyle. They were also asked to fast for a minimum of 6 h, during which time they were instructed to drink only water, and to attend for various measurements (see below) at a prespecified time between 08:00 and 18:00 hours. All men were asked to provide a blood sample, which was collected using the Sarstedt Monovette system (Sarstedt, Numbrecht, Germany). The samples were frozen and stored at –20°C on the day of collection and transferred in batches for storage at –70°C until analysis, which was carried out after no more than one freeze–thaw cycle. The men were asked whether a doctor had ever told them that they had angina or MI (heart attack, coronary thrombosis) or stroke, and to take any medication they were taking to the examination session. A total of 4252 men (77% of survivors) underwent examination and 4094 provided blood samples.

### Cardiovascular risk factors

Anthropometric measurements, including body weight, height and waist circumference, were taken. Participants were measured standing in light clothing without shoes. Height was measured using a Harpenden stadiometer (Holtain, Crymych, UK) to the last complete 0.1 cm, and weight was measured using a Soehnle digital electronic scale (Soehnle, Murrhardt, Germany) to the last complete 0.1 kg. BMI (weight/height^2^ [kg/m^2^]) was calculated for each participant. Waist circumference was measured in duplicate using an insertion tape (CMS, London, UK) at the midpoint between the iliac crest and the lower rib margins measured at the sides. Details of measurements and classification methods for smoking status, physical activity, social class and BP have been previously described [21, 22].

Moderate drinking was defined as 21–34 drinks/week (3–4 drinks/day), while heavy drinking was defined as those reporting drinking ≥35 drinks/week (≥5 drinks/day). Plasma glucose was measured with a glucose oxidase method using a Falcor 600 automated analyser (A. Menarini Diagnostics, Wokingham, UK) [23]. Serum insulin was measured using an ELISA assay (R&D systems, Oxford UK) that did not cross-react with proinsulin [24]. Triacylglycerol, glucose and insulin concentrations were adjusted for the effects of fasting duration and time of day [22]. Insulin resistance was estimated according to HOMA [25]. The estimated (e)GFR (a measure of renal function) was calculated from serum creatinine using the modification of diet in renal disease equation developed by Levey et al [26]. Chronic kidney disease was defined as eGFR <60 ml min^–1^ 1.73 m^–2^. Von Willebrand factor antigen was measured using an ELISA antigen (DAKO, High Wycombe, UK). C-reactive protein (CRP) was assayed by ultra-sensitive nephelometry (Dade Behring, Milton Keynes, UK).

### Prevalent diabetes

Men with a doctor’s diagnosis of diabetes, those with fasting glucose of ≥7 mmol/l (WHO criteria) or those with HbA_1c_ ≥6.5% (48 mmol/mol) were considered to have prevalent diabetes [27].

### Copeptin and N-terminal pro B-type natriuretic peptide (NT-proBNP)

Serum levels of copeptin and NT-proBNP were measured using manufacturers’ calibrators and controls in accordance with their instructions. NT-proBNP was determined using the Elecsys 2010 electrochemiluminescence method (Roche Diagnostics, Burgess Hill, UK) [28]. The lower limit of sensitivity was 5 ng/l. Low control CV was 6.7% and high control CV was 4.9%. Copeptin was measured using an ultra-sensitive method on a BRAHMS Kryptor Compact Plus (BRAHMS, Bottisham, UK). The lower limit of sensitivity was 0.9 pmol/l. Low control CV was 4.7% and high control CV was 4.6%. We examined copeptin levels by the time of examination. Although copeptin levels were highest at 13:00 hours (geometric mean 4.95 pmol/l, interquartile range [IQR] 2.76–8.11 pmol/l), there was no consistent significant diurnal variation throughout the day. For example, the mean copeptin level between 09:00 and 10:00 hours (3.97 pmol/l [IQR 2.50–6.58 pmol/l]) was similar to that between 12:00 and 13:00 hours (3.94 pmol/l [IQR 2.49–6.49 pmol/l]) and between 17:00 and 18:00 hours (3.94 pmol/l [IQR 2.61–6.32 pmol/l]).

### Follow-up

All of the men were followed for cardiovascular morbidity from the initial examination (1978–1980), and follow-up was achieved for 99% of the cohort [29]. In the present analyses, all-cause mortality and morbidity events were based on follow-up from re-screening in 1998–2000 at a mean age of 60–79 years to July 2012, a mean follow-up period of 13 years (range 12–14 years). Information on death was collected through National Health Service registers, in which the participants were flagged for follow-up [30]. During the follow-up period, changing diagnostic criteria were taken into account by using both ICD-9 and ICD-10 codes. For the present paper, CHD deaths were defined as ICD-9 codes 410–414 or ICD-10 codes I20–I25. Stroke deaths were defined as ICD-9 codes 430–438 or ICD-10 codes I60–I69. Non-fatal stroke events were defined as those that resulted in a neurological deficit that was present for more than 24 h. Evidence regarding non-fatal stroke and MI events was obtained by ongoing reports from general practitioners, by biennial reviews of patients’ practice records (including hospital and clinic correspondence) through to the end of the study period and from repeated questionnaires sent to surviving participants after the initial examination. Pre-existing CVD was defined as a history of a doctor’s diagnosis of MI or stroke. Patient recall of a doctor’s diagnosis of CHD has been shown to be a valid measure of recording disease in this study population [31]; the k statistic for comparing record reviews with patients’ recall of CHD was 0.82 [31].

### Statistical methods

The distribution of copeptin levels was skewed and natural log transformation was therefore used. Cox’s proportional hazards model was used to assess multivariate-adjusted HRs (relative risk) in a comparison of three copeptin groups (<3.35, 3.35–6.95 and ≥6.96 pmol/l), as well as in a 1 unit increase in log_*e*_ copeptin. As men with diabetes showed significantly higher levels of copeptin than men without diabetes (mean 6.96 pmol/l [IQR 2.90–8.63 pmol/l] vs 3.34 pmol/l [IQR 2.40–5.88 pmol/l], respectively), the cut-offs for the three groups were based on the tertile distribution in men with diabetes to achieve sufficient numbers in each group and then applied to those without diabetes. Thus, the number of men in each of the three groups was not equally distributed in those without diabetes.

In multivariate analyses, smoking (never, long-term ex-smokers [>15 years], recent ex-smokers [<15 years] and current smokers), social class (manual vs non-manual work), physical activity (four groups), heavy drinking (yes/no), prevalent MI and prevalent stroke were fitted as categorical variables; CRP, eGFR, BMI, systolic BP, HDL-cholesterol, HOMA-IR, blood glucose and NT-proBNP were fitted as continuous variables. Adjustment for diurnal variation was carried out by fitting the time of examination as three groups: morning (9:00–13:00 hours), lunchtime (13:00 hours) and afternoon (14:00–18:00 hours). A test for interaction was carried out by fitting the interaction term ‘log_*e*_ copeptin × prevalent diabetes (yes/no)’ into the model. A sensitivity analysis was performed excluding men with prevalent MI or stroke. *p* < 0.05 was used to indicate statistical significance.

### Study sample

Copeptin measurements were available for 3663 men. Because heart failure is known to raise AVP (copeptin) and copeptin may be a marker of the severity of heart failure [32], we excluded 127 men with a history of heart failure, leaving 3536 men for analysis.

## Results

During the mean follow-up period of 13 years, the 3536 men without heart failure experienced 437 major CHD events (11.3/1000 person-years) and 323 major stroke events (8.6/1000 person-years), with 497 deaths from cardiovascular causes.

Table 1 shows baseline characteristics by the three copeptin groups in men with and without diabetes. Copeptin was strongly related to BMI, renal function, HOMA-IR and NT-proBNP in both the diabetic and non-diabetic groups. The prevalence of stroke and MI was highest in those with elevated copeptin.

**Table 1:** Baseline characteristics (1998–2000) in 3536 men without prevalent heart failure according to diabetes status and serum copeptin level

Variable	No diabetes				Diabetes			
	Copeptin (pmol/l)^a^				Copeptin (pmol/l)^a^			
	<3.35 (*n* = 1376)	3.35–6.95 (*n* = 1151)	≥6.96 (*n* = 581)	*p*_trend_^b^	<3.35 (*n* = 143)	3.35–6.95 (*n* = 142)	≥6.96 (*n* = 143)	*p*_trend_^b^
Age (years)	68.0 ± 5.4	68.8 ± 5.7	69.4 ± 5.4	<0.001	68.5 ± 5.3	68.9 ± 5.0	69.3 ± 5.3	0.12
BMI (kg/m^2^)	26.1 ± 3.3	26.9 ± 3.7	27.5 ± 3.8	<0.001	27.3 ± 3.6	28.1 ± 3.7	29.3 ± 5.0	<0.001
Physically inactive (%)	29.6	31.8	39.8	<0.001	40.0	47.0	50.0	0.09
Current smoking (%)	11.4	13.7	13.3	0.11	7.0	12.8	15.4	0.02
Manual work (%)	50.8	54.2	61.1	<0.001	55.9	56.3	55.0	0.98
Moderate/heavy drinking (%)	9.8	14.1	11.5	0.02	16.8	12.7	14.0	0.42
On antihypertensive treatment (%)	26.3	29.6	40.1	<0.001	42.0	47.2	60.1	0.004
On ACE inhibitors (%)	5.1	7.4	9.5	0.0003	17.5	17.7	28.8	0.04
Stroke (%)	4.5	5.6	6.7	0.04	7.0	6.3	13.3	0.11
MI (%)	5.3	7.2	8.1	0.01	6.3	4.9	9.1	0.47
Systolic BP (mmHg)	146.4 ± 23.2	150.0 ± 23.9	151.2 ± 24.4	<0.001	154.4 ± 25.9	156.3 ± 24.7	151.7 ± 25.3	0.52
Total cholesterol (mmol/l)	6.03 ± 1.04	6.05 ± 1.08	5.97 ± 1.11	0.55	5.88 ± 1.15	5.79 ± 1.45	5.87 ± 1.09	0.82
HDL-cholesterol (mmol/l)	1.35 ± 0.33	1.34 ± 0.34	1.28 ± 0.34	0.001	1.26 ± 0.30	1.25 ± 0.32	1.19 ± 0.34	0.14
Glucose (mmol/l)	5.53 (5.21–5.90)	5.53 (5.23–5.91)	5.58 (5.22–5.95)	0.16	8.17 (6.50–10.10)	8.00 (6.70–8.90)	9.21 (7.16–9.21)	0.02
HbA_1c_ (%)	4.78 ± 0.54	4.81 ± 0.54	4.84 ± 0.57	0.04	6.36 ± 1.29	6.22 ± 1.47	6.69 ± 1.64	0.14
HbA_1c_ (mmol/mol)	28.8 ± 5.9	29.1 ± 5.9	29.4 ± 6.3		46.0 ± 14.1	44.5 ± 16.1	49.7 ± 17.9	
Log_*e*_ HOMA-IR	0.58 ± 0.58	0.68 ± 0.58	0.82 ± 0.61	<0.001	1.59 ± 1.07	1.58 ± 0.95	2.01 ± 1.01	0.002
Serum sodium (mmol/l)	139.4 ± 2.7	139.8 ± 2.7	140.3 ± 2.3	0.04	138.5 ± 3.0	138.9 ± 2.9	139.0 ± 3.2	0.11
eGFR (ml min^–1^ 1.73 m^–2^)	75.8 ± 11.5	72.9 ± 12.2	66.7 ± 14.0	<0.001	74.9 ± 12.0	72.4 ± 12.2	65.6 ± 13.6	<0.001
eGFR <60 ml min^–1^ 1.73 m^–2^ (%)	7.1	13.0	28.9	<0.001	11.2	17.6	29.4	0.002
CRP (mg/l)	1.38 (0.66–2.71)	1.73 (0.86–3.46)	2.29 (1.04–4.96)	<0.001	1.68 (0.84–2.97)	2.18 (1.02–3.91)	2.85 (1.30–6.29)	<0.001
von Willebrand factor (IU/l)	1300.0 ± 420.0	1390.2 ± 430.6	1500.7 ± 480.2	<0.001	1380.1 ± 490.1	1480.1 ± 480.6	1690.8 ± 50.4	<0.001
NT-proBNP (ng/l)	83.9 (43–78)	98.5 (46–190)	133.0 (55–284)	<0.001	80.6 (35–181)	111.1 (53–221)	144.0 (53–325)	<0.001

Data are mean ± SD or geometric mean (IQR)

^a^Cut-offs based on the tertile distribution in men with diabetes

^b^Across groups

### Copeptin and incident CVD in men without diabetes

Elevated copeptin was associated with a significantly increased risk of CHD events after adjustment for age (Table 2). Further adjustment for CVD risk factors including smoking, physical activity, social class, BMI, HDL-C, systolic BP, antihypertensive treatment, eGFR, prevalent MI, prevalent stroke, CRP, HOMA-IR and blood glucose (Model 2) attenuated the association, although it remained statistically significant. However, the association was markedly reduced after the exclusion of men with prevalent CVD (MI or stroke). There was no significant association between copeptin and incident stroke or CVD mortality after adjustment for these CVD risk factors in Model 2. Additional adjustment for diurnal variations made little difference to the findings.

**Table 2:** Incidence rates/1000 person-years and adjusted HRs (95% CIs) for CVD endpoints by copeptin levels in 3108 men with no prevalent diabetes and no heart failure

Incidence	Copeptin (pmol/l)^a^			1 unit increase in log_*e*_ copeptin	*p* trend
	<3.35 (*n* = 1376)	3.35–6.95 (*n* = 1151)	≥6.96 (*n* = 581)		
CHD (*n* = 370)					
Rate/1000 person-years (*n*)	8.5 (134)	11.4 (146)	15.2 (90)		
Age-adjusted HR	1.00	1.27 (1.01, 1.61)	1.68 (1.28, 2.19)	1.23 (1.00, 1.40)	0.002
Model 1	1.00	1.24 (0.97, 1.57)	1.45 (1.10, 1.92)	1.15 (1.01, 1.30)	0.04
Model 2	1.00	1.21 (0.95, 1.55)	1.36 (1.01, 1.83)	1.12 (0.98, 1.27)	0.10
Model 2a	1.00	1.24 (0.94, 1.62)	1.22 (0.87, 1.71)	1.04 (0.91, 1.19)	0.54
Stroke (*n* = 272)					
Rate/1000 person-years (*n*)	7.2 (111)	8.2 (101)	10.5 (60)		
Age-adjusted HR	1.00	1.07 (0.81, 1.40)	1.37 (1.00, 1.84)	1.12 (0.97, 1.29)	0.13
Model 1	1.00	1.00 (0.76, 1.32)	1.16 (0.84, 1.62)	1.03 (0.90, 1.17)	0.65
Model 2	1.00	0.99 (0.75, 1.31)	1.13 (0.80, 1.59)	1.03 (0.90, 1.17)	0.71
Model 2a	1.00	1.12 (0.82, 1.53)	1.26 (0.79, 1.71)	1.05 (0.90, 1.24)	0.52
CVD death (*n* = 419)					
Rate/1000 person-years (*n*)	9.2 (144)	13.2 (168)	18.1 (107)		
Age-adjusted HR	1.00	1.31 (1.05, 1.64)	1.82 (1.41, 2.33)	1.29 (1.14, 1.46)	0.005
Model 1	1.00	1.18 (0.93, 1.48)	1.43 (1.10, 1.86)	1.13 (1.00, 1.28)	0.05
Model 2	1.00	1.15 (0.91, 1.45)	1.28 (0.97, 1.69)	1.08 (0.95, 1.22)	0.22
Model 2a	1.00	1.19 (0.96, 1.60)	1.02 (0.73, 1.43)	0.97 (0.86, 1.11)	0.62

^a^Cut-offs based on the tertile distribution in men with diabetes

Model 1: adjusted for age, smoking, physical activity, heavy drinking, social class, BMI, HDL-C, systolic BP, antihypertensive treatment, prevalent MI and prevalent stroke

Model 2: Model 1 + CRP + HOMA-IR + blood glucose + eGFR

Model 2a: adjusted for Model 1 and excluding men with prevalent MI/stroke (*n* = 353; 11.4%)

### Copeptin and incident CVD in men with diabetes

In contrast to men without diabetes, copeptin was significantly associated with incident stroke and CVD mortality among men with diabetes, even after adjustment for cardiovascular risk factors and upon the exclusion of men with prevalent CVD (Table 3). Additional adjustment for the use of ACE inhibitors made little difference to these findings. A formal test for interaction confirmed a significant difference in the relationship between copeptin and risk of stroke and CVD between men with and without diabetes after the exclusion of men with MI or stroke (*p* = 0.01 and *p* = 0.02, respectively).

**Table 3:** Incidence rates/1000 person-years and adjusted HRs (95% CIs) for CVD endpoints by copeptin levels in 428 men with prevalent diabetes and no heart failure

Incidence	Copeptin (pmol/l)^a^			1 unit increase in log_*e*_ copeptin	*p* trend
	<3.35 (*n* = 143)	3.35–6.95 (*n* = 142)	≥6.96 (*n* = 143)		
CHD (*n* = 67)					
Rate/1000 person-years (*n*)	12.5 (20)	13.6 (19)	22.5 (28)		
Age-adjusted HR	1.00	1.11 (0.59, 2.05)	1.85 (1.04, 3.29)	1.17 (0.86, 1.59)	0.33
Model 1	1.00	0.95 (0.49, 1.82)	1.59 (0.83, 3.04)	1.02 (0.77, 1.36)	0.88
Model 2	1.00	0.88 (0.45, 1.73)	1.21 (0.60, 2.42)	0.94 (0.72, 1.22)	0.63
Model 3	1.00	0.86 (0.43, 1.68)	1.18 (0.59, 2.35)	0.94 (0.73, 1.20)	0.59
Model 3a	1.00	0.76 (0.36, 1.60)	0.99 (0.47, 2.09)	1.00 (0.75, 1.33)	0.99
Stroke (*n* = 51)					
Rate/1000 person-years (*n*)	7.7 (12)	11.2 (15)	20.5 (24)		
Age-adjusted HR	1.00	1.49 (0.70, 3.18)	2.69 (1.34, 5.40)	1.87 (1.29, 2.71)	0.001
Model 1	1.00	1.96 (0.88, 4.36)	2.34 (1.11, 4.91)	1.62 (1.11, 2.37)	0.01
Model 2	1.00	2.11 (0.93, 4.76)	2.22 (1.01, 4.89)	1.63 (1.07, 2.48)	0.02
Model 3	1.00	2.16 (0.93, 4.98)	2.34 (1.04, 5.27)	1.65 (1.06, 2.55)	0.03
Model 3a	1.00	2.03 (0.71, 5.81)	2.97 (1.09, 8.12)	1.73 (0.98, 3.03)	0.05
CVD death (*n* = 78)					
Rate/1000 person-years (*n*)	10.0 (16)	17.8 (25)	29.7 (37)		
Age-adjusted HR	1.00	1.77 (0.94, 3.31)	2.78 (1.54, 4.99)	1.79 (1.33, 2.39)	<0.0001
Model 1	1.00	1.67 (0.88, 3.18)	2.99 (1.57, 5.71)	1.62 (1.18, 2.23)	0.003
Model 2	1.00	1.65 (0.86, 3.16)	2.49 (1.27, 4.90)	1.47 (1.06, 2.04)	0.02
Model 3	1.00	1.55 (0.80, 3.03)	2.21 (1.12, 4.36)	1.34 (0.98, 1.84)	0.06
Model 3a	1.00	1.76 (0.80, 3.91)	3.03 (1.39, 6.62)	1.92 (1.23, 2.98)	0.004

^a^Cut-offs based on the tertile distribution in men with diabetes

Model 1: adjusted for age, smoking, heavy drinking, physical activity, social class, BMI, HDL-C, systolic BP, antihypertensive treatment, prevalent MI and prevalent stroke

Model 2: Model 1 + CRP + HOMA-IR + blood glucose + eGFR

Model 3: Model 2 + NT-proBNP

Model 3a: Model 3, excluding men with prevalent MI/stroke (*n* = 61; 14.3%)

No association was seen between copeptin and the risk of major CHD events (fatal or non-fatal) in men with diabetes after adjustment for established cardiovascular risk factors (Table 3). In contrast to the significant interaction seen for stroke and CVD mortality, there was no significant interaction for CHD events between men with and without diabetes (*p* = 0.81). Additional adjustment for diurnal variation made no material difference to the findings. When examined in relation to non-fatal (*n* = 19) and fatal (*n* = 48) CHD events, a non-significant inverse association between copeptin levels and non-fatal events was seen, and a positive association was seen with fatal events after adjustment for factors in Model 3, although the numbers were small and the increased risk was not significant (adjusted HR 1.68 [95% CI 0.72, 3.92]; top third vs lowest third). Although CHD deaths accounted for more than 50% of all CVD deaths (*n* = 48/78), the increased risk of CVD mortality was largely due to stroke or other CVD deaths (adjusted HR 4.64 [95% CI 1.27, 16.74]).

## Discussion

In this study of older men, copeptin was significantly associated with incident stroke and CVD mortality in men with diabetes, after adjustment for established CVD risk factors and following the exclusion of men with MI or stroke, and in keeping with the markedly elevated copeptin levels in this patient group. In contrast, we did not find any significant association between copeptin and incident stroke or CVD mortality in men without diabetes after adjustment for established CVD risk factors. Copeptin was positively associated with incident CHD in men both with and without diabetes, but these associations were to a large extent associated with pre-existing CVD as the exclusion of individuals with MI/stroke attenuated the association in both groups. Our findings confirm previous studies reporting an association between copeptin and CVD in diabetes [14–18], and extend the findings to stroke incidence in individuals both with and without diabetes in the general population. We were able to take into account a wide range of CVD risk markers, including insulin resistance, inflammation, renal function and NT-proBNP.

### Diabetes status, copeptin and CVD

Our findings extend those of a recent report in which copeptin predicted CVD endpoints in individuals with diabetes only [18]. Vasopressin levels are increased in patients with diabetes, although the cause of this is still unclear [10]. Although copeptin has previously been associated with CVD or stroke in specific subgroups of patients with diabetes and MI [16] or on haemodialysis [15], we are not aware of any other studies that have examined the association between copeptin and stroke in the general population of older people with diabetes. In this study, we observed a strong association between copeptin and stroke events, but not CHD events, after taking into account a wide range of vascular risk factors and upon exclusion of those with MI or stroke. We also observed a non-significant positive association between copeptin and CHD in the diabetes group. Although the lack of significance in the diabetes group might be due to small numbers, the positive association with CHD was markedly attenuated upon exclusion of those with MI or stroke, and a similar pattern of attenuation was also seen in participants without diabetes. Thus the increased risk of CHD associated with copeptin seen in both the diabetic and non-diabetic groups appears to be largely due to pre-existing CVD. This is consistent with previous reports that have shown copeptin to predict CVD specifically in those with coronary artery disease [33, 34].

Similar findings have also been reported in the German Diabetes and Dialysis Study, in which copeptin predicted stroke but not MI in diabetes patients undergoing haemodialysis [15]. In the Malmo Diet and Cancer Study [18] a positive association was seen with CHD in the diabetes group, which included individuals who developed diabetes during follow-up as well as prevalent diabetes cases. We did not include incident diabetes in our population of participants with diabetes. However, when the Malmo Diet and Cancer Study restricted its analysis to prevalent diabetes only, there was no association between copeptin and CHD events, similar to our findings.

### Copeptin and risk of major stroke events

Brain injury, including ischaemia, causes the release of stress hormones such as AVP [35]. It is increasingly being recognised that copeptin predicts adverse outcomes in patients with stroke [36]. However, reports on the association between copeptin and incident stroke in the general population are lacking. Our findings show that copeptin is associated with the development of stroke, even in diabetes patients without prevalent stroke. Whether the relationship between copeptin and stroke risk in diabetes is causal is uncertain, but it was not explained by conventional risk factors for stroke, insulin resistance or NT-proBNP. Renal dysfunction has been associated with an increased risk of stroke [37] and AVP is known to be associated with declining kidney function in patients with diabetes [38]. However, the association between copeptin and stroke was seen after adjustment for eGFR and even after the exclusion of men with chronic kidney disease. Population-based studies have shown copeptin to be strongly associated with microalbuminuria [39], suggesting a role for the AVP system in the development of albuminuria, which has been linked to incident stroke in diabetes as well as in the general population [39, 40]. This may be a consequence of the antidiuretic effect of vasopressin, which leads to increased urinary albumin excretion, an effect mediated by vasopressin 2 receptors [38, 41]. Thus, the association between copeptin, which is a marker of endogenous vasopressin secretion, and incident stroke may be linked to increased albuminuria.

Our finding of an association between copeptin and CVD mortality and stroke events in individuals with diabetes, but not in those without diabetes, is of potential relevance to the recent findings of the EMPA-REG OUTCOME trial, particularly given prior associations of elevated copeptin with renal and cardiac pathology, heart failure and CVD mortality. EMPA-REG OUTCOME, which investigated the effects of the sodium-glucose transporter 2 (SGLT2) inhibitor empagliflozin on cardiovascular morbidity and mortality in patients with type 2 diabetes at high cardiovascular risk, demonstrated a substantial reduction in CVD death and heart failure hospitalisations with empagliflozin but no effect on non-fatal CVD events [42]. One may speculate that many diabetes patients with CVD have subclinical fluid overload, making them exquisitely sensitive to fluid balance changes. Notably, SGLT2 inhibitors promote glycosuria by lowering the renal glucose reabsorption threshold, whereas AVP promotes water reabsorption through stimulation of V_2_ receptors, and increased levels of AVP appear to limit glucose-induced water loss in patients with diabetes [43]. These findings suggest that SGLT2 inhibitors should lessen copeptin levels in line with improved fluid balance and cardiac and renal function in diabetes patients with CVD, a hypothesis that should be directly testable in the near future.

### Strengths and limitations

The strengths and limitations of this study require consideration. This is the first study to examine the association between copeptin and incident stroke in a general population of older men with and without diabetes, who constitute a high-risk group for CVD. The population of the British Regional Heart Study, though moderate in size, is socially representative of the UK and follow-up rates are exceptionally high. Ascertainment of CHD death and MI in this study was based on standard methods, and both CHD mortality and MI incidence rates correspond closely with national data. We were able to take into account a wide range of cardiovascular risk factors, including markers of inflammation and cardiac markers. However, our study was based on an older, predominantly white European, male population, so the results cannot be generalised directly to women, younger men or other ethnic groups; the results therefore need to be substantiated in other studies and in different population groups. Furthermore, our findings are based on a single measurement at the baseline re-examination rather than on repeated assessments, which may have led to an underestimation of the true strength of associations. In addition, we did not have information on microalbuminuria. Although we did not have information on the type of stroke for all men, 85% of stroke cases in Great Britain are caused by cerebral ischaemia [44].

### Conclusion

In this study, we found a significant association between copeptin and subsequent stroke risk and CVD mortality in men with diabetes, but not in men without diabetes, with those with diabetes also having substantially elevated copeptin levels. Future studies are required to explore whether elevated copeptin, as a sensitive marker for AVP secretion, is just a risk marker or a potentially modifiable risk factor for CVD mortality and stroke in diabetes. The latter would suggest that suppressing vasopressin with vasopressin antagonists may have a potential role in reducing stroke and CVD mortality among individuals with diabetes.

## Abbreviations

AVP: Arginine vasopressin
CRP: C-reactive protein
CVD: Cardiovascular disease
eGFR: Estimated GFR
MI: Myocardial infarction
NT-proBNP: N-terminal pro B-type natriuretic peptide
SGLT2: Sodium-glucose transporter 2
